# Sieve-based coreference resolution enhances semi-supervised learning model for chemical-induced disease relation extraction

**DOI:** 10.1093/database/baw102

**Published:** 2016-07-26

**Authors:** Hoang-Quynh Le, Mai-Vu Tran, Thanh Hai Dang, Quang-Thuy Ha, Nigel Collier

**Affiliations:** ^1^Faculty of Information Technology, VNU University of Engineering and Technology, Hanoi, Vietnam. Building E3, 144 Xuan Thuy str., Cau Giay dist., Hanoi, Vietnam. Postal code: 100000; ^2^Department of Theoretical and Applied Linguistics, University of Cambridge, Cambridge, UK

## Abstract

The BioCreative V chemical-disease relation (CDR) track was proposed to accelerate the progress of text mining in facilitating integrative understanding of chemicals, diseases and their relations. In this article, we describe an extension of our system (namely UET-CAM) that participated in the BioCreative V CDR. The original UET-CAM system’s performance was ranked fourth among 18 participating systems by the BioCreative CDR track committee. In the Disease Named Entity Recognition and Normalization (DNER) phase, our system employed joint inference (decoding) with a perceptron-based named entity recognizer (NER) and a back-off model with Semantic Supervised Indexing and Skip-gram for named entity normalization. In the chemical-induced disease (CID) relation extraction phase, we proposed a pipeline that includes a coreference resolution module and a Support Vector Machine relation extraction model. The former module utilized a multi-pass sieve to extend entity recall. In this article, the UET-CAM system was improved by adding a ‘silver’ CID corpus to train the prediction model. This silver standard corpus of more than 50 thousand sentences was automatically built based on the Comparative Toxicogenomics Database (CTD) database. We evaluated our method on the CDR test set. Results showed that our system could reach the state of the art performance with F1 of 82.44 for the DNER task and 58.90 for the CID task. Analysis demonstrated substantial benefits of both the multi-pass sieve coreference resolution method (F1 + 4.13%) and the silver CID corpus (F1 +7.3%).

**Database URL**: SilverCID–The silver-standard corpus for CID relation extraction is freely online available at: https://zenodo.org/record/34530 (doi:10.5281/zenodo.34530).

## Introduction

A survey of PubMed users’ search behavior showed that diseases and chemicals were two of the most frequently requested entities by PubMed users worldwide: diseases appeared in 20% of queries and chemicals in 11% (1). These two entities are central to several topics such as developing drugs for therapeutics, discovering adverse drug reactions (ADRs) as well as chemical safety/toxicity among patient groups and facilitating hypothesis discovery for new pharmaceutical substances. As a consequence, extracting chemical-disease relations (CDR) from unstructured free text has become an important field in biomedical text mining.

In recent years, there has been an increased focus in research on capturing disease and chemical relations (e.g. drug-side-effect relations) from biomedical literature text. The Comparative Toxicogenomics Database (CTD) has been a notable target of many studies. The CTD is a manually curated database that promotes understanding about the effects of environmental chemicals (e.g. arsenic, heavy metals and dioxins) on human health (2). As of June 2015, the CTD database had 1 842 746 chemical–disease associations. Due to the high cost of manual curation and the rapid growth of the biomedical literature, a number of researchers have attempted to extract chemical—disease relations or drug side effects automatically. The simplest class of approaches is based on the co-occurrence statistics of chemical and disease entities, i.e. if two entities are mentioned together in the same sentence or abstract, they are probably related. Chen *et al.* (3) used this method to identify and rank associations between eight diseases and relevant drugs. This approach tends to achieve high recall, but low precision and fails to distinguish the chemical-induced disease (CID) relations from other relations that commonly occur between chemicals and diseases. Knowledge-based approaches were also successfully applied for the ADR extraction (4, 5). They, however, demands the time-consuming and labor-intensive manual compilation of huge knowledge (in terms of rules as in 4 or a three-tier hierarchical graph as in 5), which results from the wide variety of contexts in which relations can occur. These approaches, therefore, tend to suffer from the low recall. Other approaches are based on automated machine learning techniques, such as Support Vector Machines (SVMs) (6) and decision trees (7). Their performance, however, has still been limited, which is mainly due to the lack of a substantial data set for training. Moreover, the variety of abundant ADR syntaxes as well as a failure to resolve inter-sentence alternative entity-mentions also hampers the performance.

To accelerate the progress, BioCreative V proposed a challenge task for automatic extraction of CDRs (8, 9).

The CDR challenge has two sub-tasks:

(A) Disease Named Entity Recognition (DNER). This task includes automatic recognition of disease mentions (named entity recognition, NER) in PubMed abstracts and assignment of Medical Subject Heading (MeSH, 10) identifiers to these mentions named entity normalization (NEN).
(B) CID relation extraction. Participating systems were provided with raw text from PubMed articles as input and asked to return a list of <chemical, disease> pairs. In which, chemicals and diseases are normalized concepts that participate in a CID relation.

**Figure 1.**
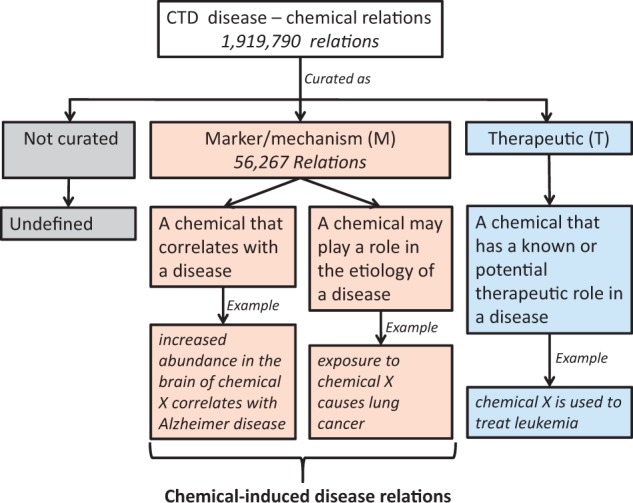
Analysis of the direct evidence field in the CTD database.

In these challenge tasks, diseases were annotated using the ‘Diseases’ [C] branch of MeSH 2015, including diseases, disorders, signs and symptoms; chemical terminologies were annotated using the ‘Drugs and Chemicals’ [D] branch of MeSH 2015. The CID relations can be marked as ‘marker/mechanism’ in the CTD database. There are two types of such relationships: (i) biomarker relations between a chemical and disease indicating that the chemical correlates with the disease and (ii) putative mechanistic relationships between a chemical and disease indicating that the chemical may play a role in the etiology of the disease (see Figure 1).

As a team participating in the CDR challenge, we proposed a modular system that handled the DNER and CID tasks separately. For the DNER as the first phase, we proposed a method for combining several state-of-the-art word-embedding techniques in the NEN module in order to take advantages of both the gold standard annotated corpus and large scale unlabeled data. The NEN and NER modules were then combined into a joint inference model to boost performance and reduce noise. For the second phase, the CID task exposed many challenges such as (i) complex grammatical structures, (ii) entities that belong to a relation may appear not only in a single sentence but also in multiple sentences, in which they are often mentioned coreferentially or using different forms, (iii) entities being expressed in MeSH IDs instead of of free-text forms. To overcome these challenges, a traditional machine learning model for relation extraction, which is based only on explicit mentions of entities in a single sentence, will not be adequate. We thus had to employ a coreference module along with a SVM-based relation extraction module as the central core. The intention of using the coreference module was to extend system recall on disease/chemical mentions, then to convert inter-sentence relations to intra-sentence relations. Additionally, in order to exploit as much useful information as possible from the literature, we built a silver-standard corpus (namely ‘SilverCID’) for training the DNER average perceptron model and the SVM intra-sentence relation extraction model. This corpus was a carefully selected sub-set of citations in the CTD database and totally disjoint from the targets in the testing set. In addition, we explored the benefit of using a large-scale feature set to handle the variety of CTD relation mentions.

The novel contributions of this article are as follows: (i) we proposed a DNER model that was based on the joint inference between an averaged perceptron NER model and a NEN pipeline of two phases, i.e. Supervised Semantic Indexing (SSI) followed by a skip-gram model; (ii) we demonstrated the benefit of our automatically built SilverCID corpus (a sentence-level corpus) for the CID relation extraction; (iii) we presented evidence for the efficacy of using the multi-pass sieve in the CID relation extraction task and (iv) we demonstrated the strength of the rich feature set (see section SVM-based intra-sentence relation extraction and Table 2 for more details) for CID relation extraction.

## Materials and Methods

### Data set

Our experiments were conducted on the BioCreative V CDR data. In order to take advantage of the CTD database, we also built a SilverCID corpus from PubMed articles that were cited in the CTD database but which did not appear in the BioCreative CDR track data set.

#### BioCreative CDR track data set

To assist the development and assessment of participating CDR systems, the BioCreative V workshop organizers created an annotated text corpus that consists of expert annotations for all chemicals, diseases, and their CID relations. This corpus contained a total of 1500 PubMed articles that were separated into three sub-sets, each of 500 for the training, development and test set (the details are shown on Table 1). Following the data survey of BioCreative (9), of these 1500 articles, 1400 were selected from an existing CTD-Pfizer data set that had been jointly curated via a previous collaboration between CTD and Pfizer (11). The remaining 100 articles contained newly curated data and were incorporated into the test set.

**Table 1.** Summary of the CDR track data set

Data set	Articles	Chemical		Disease		CID
		Men	ID	Men	ID	
Training	500	5203	1467	4182	1965	1038
Development	500	5347	1507	4244	1865	1012
Test	500	5385	1435	4424	1988	1066

Men, Mention; CID, CID relations.

#### SilverCID corpus

The CTD (2) is a robust, publicly available database that aims to advance understanding about how environmental exposures affect human health. Chemicals in the CTD come from the chemical subset of MeSH. The CTD’s disease vocabulary is a modified subset of descriptors from the ‘Diseases’ category of MeSH, combined with genetic disorders from the Online Mendelian Inheritance in Man (OMIM) database (12).

In > 28 million CTD toxicogenomic relationships, there are 1 919  790 disease-chemical relations (curated or inferred via CTD-curated chemical–gene interaction) (October 2015). There are several types of relations between diseases and chemicals, which may be described within the ‘Direct Evidence’ field of the CTD database. This field has two labels *M* and *T*, in which the label *M* indicates that a chemical can correlate with a disease or can be the etiology of a disease (Figure 1). Relations curated as *M*, therefore, are more likely to be CID relations.

**Figure 2.**
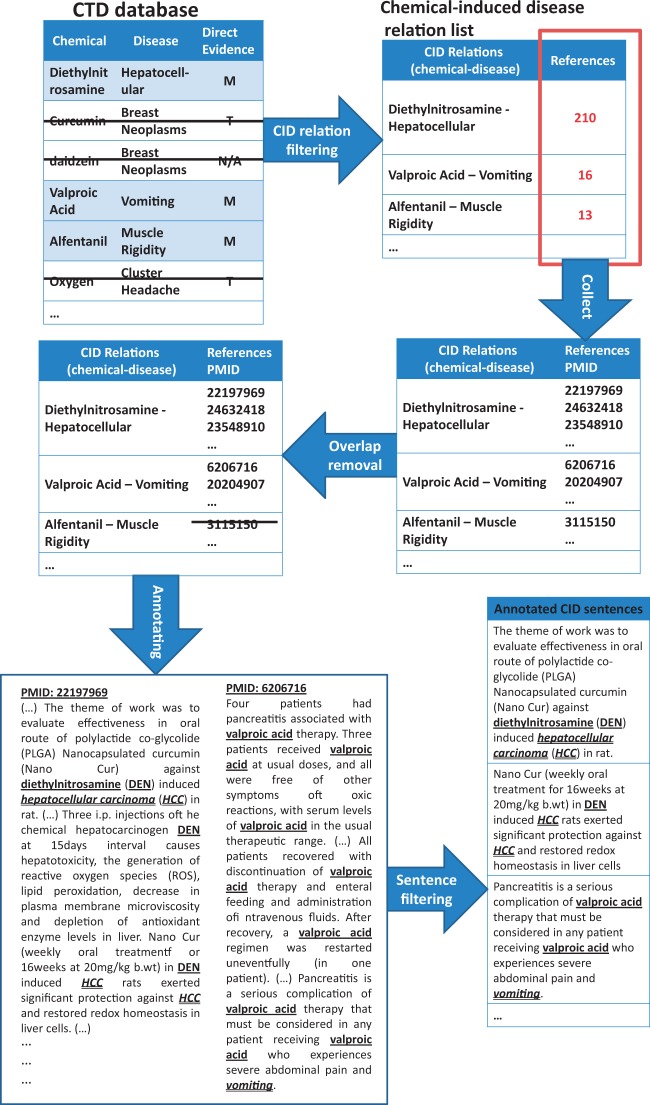
An example of constructing silverCID corpus.

Moreover, we observed that if two entities that participated in a relation appear in the same sentence, it is highly probable that this sentence contains the grammatical relation that we were considering. Taking into account these two observations, a silver standard CID corpus, SilverCID, was constructed using the CTD database and PubMed according to five steps (Figure 2 gives an example of how the SilverCID was constructed):

Step 1 (Relation filtering*)*: CID relations in the CTD database were filtered using information from the ‘Direct evidence’ field. Only relations marked as ‘M’ (marker/mechanism) were chosen.

Step 2 (Collecting): We collected PubMed abstracts from the reference list of the relations that had been chosen in Step 1. This reference list was provided by the CTD database.

Step 3 (Overlap removal): To avoid overlap between the SilverCID corpus and the CDR test set, we removed all the PubMeb abstracts which appeared in the CDR track data set to ensure a fair evaluation of the SilverCID’s contribution.

Step 4 (Annotating): For each relation that had been chosen in Step 1, all disease and chemical mentions in its referring PubMed articles were automatically annotated.

Step 5 (Sentence filtering): Sentences in the abstracts that remained after Step 4 were kept for downstream works if they contained both chemical and disease entities, which may participate in a CID relation. Sentences that did not contain any entity or contained only one entity were removed.

Two novel aspects that makes our SilverCID corpus different from other resources are (i) it was built automatically and (ii) it is a sentence-level corpus (i.e. a set of sentences that contains at least one intra-sentence CID relation with its participating chemical and disease entities), which covered about 60% of CID relations in the CTD database.

This data set contains 38 332 sentences, 1.25 million tokens, 48 856 chemical entities (1196 unique chemical entities), 44 744 disease entities (2098 unique disease entities) and 48 199 CID relations (12 776 unique CID relations). It is freely available online at URL: https://zenodo.org/record/34530 (doi:10.5281/zenodo.34530).

### Proposed model

**Figure 3.**
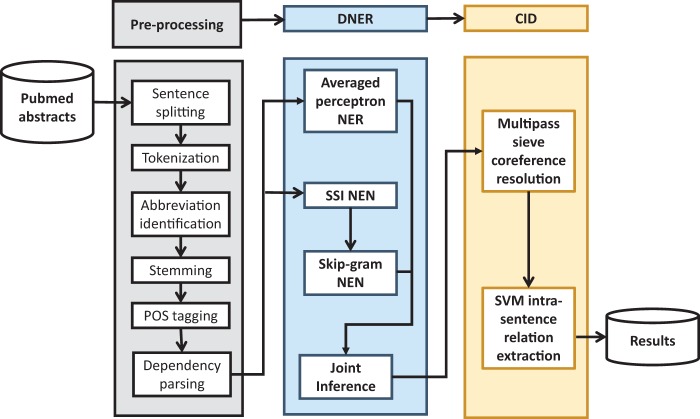
Architecture of the proposed CDR extraction system, which includes the pipeline of processing modules and material resources; boxes with dotted lines indicate sub-modules.

The overall architecture of our proposed system is described in Figure 3. Compared with our previous system in the BioCreative CDR track, the improved system used the SilverCID corpus for training in both the DNER and CID phases. The impact of this improvement on the system’s performance will be analyzed in the next sections. Pre-processing steps include sentence splitting, tokenization, abbreviation identification, stemming, POS tagging and dependency parsing (Stanford; Stanford Dependencies: http://nlp.stanford.edu/software/stanford-dependencies.shtml). The system was based on the integration of several state-of-the-art machine learning techniques in order to maximize their strengths and overcome their weaknesses.

#### Named entity recognition and normalization

This module solved the sub-task DNER. It was a joint-decoding model of a NER and NEN modules in order to boost performance and reduce noises (13). The NER and NEN modules were trained separately and then decoded simultaneously.

Following reports of high level performance of the joint-inference model by Li and Ji (13) and Zhang and Clark (14), we decided to employ a structured perceptron model for NER. Its output was a set of real numbers, each in which corresponded to the weight of each class label. This output format was the same with that of the NEN model, therefore, it was suitable for joint-inference in the decoding phase. The structured perceptron was an extension of the standard perceptron for structured prediction by applying inexact search with violation-fixing update methods (15). It was trained on the CDR training, development set and SilverCID corpus with a standard lexicographic feature set: orthography features, context features, POS tagging features and dictionary (CTD) features.

The NEN module was a sequential back-off model based on two word embedding (WE) methods: SSI (16)—a supervised WE method—and skip-grams (17)—an unsupervised WE method. The SSI model was trained on the CDR training and development set to obtain a correlation matrix *W* between tokens in the training data as well as MeSH. Skip-gram is a state-of-the-art word-to-vector method that took advantage of large unlabeled data. We used an open source skip-gram model provided by NLPLab (http://evexdb.org/pmresources/vec-space-models/wikipedia-pubmed-and-PMC-w2v.bin), which was trained on all PubMed abstracts and PMC full texts (4.08 million distinct words). The output of skip-gram model was a set of word vectors of 200 dimensions, from which similarities between all word pairs were calculated. As a result, we constructed a correlation matrix that was in the same format as the output of the SSI model. Therefore, we could combine the SSI model and the skip-gram model into the back-off model. For normalizing entities, we created pairs of each entity and each MeSH concept and then processed them by the SSI and skip-gram sequential back-off model. In this regard, firstly, we implemented the SSI model to find which pairs are linked, and then processed non-linked pairs once again by the skip-gram model.

The CID subtask required the system to extract the CID relations at the abstract level. In simple cases, a CID relation might be expressed in a single sentence (intra-sentence relation), i.e. two entities that participate in a CID relation appear in the same sentences. Unfortunately, they might be expressed in multiple sentences (inter-sentence relation). Our system was based on a strategy that firstly converted inter-sentence relations to intra-sentence relations by using a coreference resolution method and then applied a machine learning model to extract them.

Our DNER system was a joint decoding model, which used a modified beam search for decoding (13, 18). In this model, we trained two separate models for NER and NEN and then decoded them simultaneously. We also proposed a new scoring function for Beam search decoding as followed (see formula 1).

$$argmax\sum_{i=1}^{n}\left(w_{NER}\left(x_{t=i},y_{t=i-1;NER}\right)+w_{NEN}\left(x_{t=i}{,x}_{t=i-1},y_{t=i-1;NER},y_{t=i;NER}\right)\right)$$

The scoring function for NEN is:

wNENxt=i,xt=i-1,yt=i-1;NER,yt=i;NER={0, if yt=i;OwNENxt=i, if{ yt=i-1;B-DS|I-DS|O and yt=i;B-CD yt=i-1;B-CD|I-CD|O and yt=i;B-DS wNEN(xt=i,xt=i-1), if{ yt=i-1;B-DS|I-DS and yt=i;I-DS yt=i-1;B-CD|I-CD and yt=i;I-CD

If *W*_NEN_*_ _<_ _W*_NEN_ (NONE)* = *threshold, re-write formula 1 to formula 3:

$$argmax\sum_{i=1}^{n}\left(w_{NER}\left(x_{t=i},y_{t=i-1;NER}\right)+w_{NEN}\left(NONE\right)\right)$$

In which, *W*_NER_ is returned from the structured perceptron model.

#### Coreference resolution

**Figure 4.**
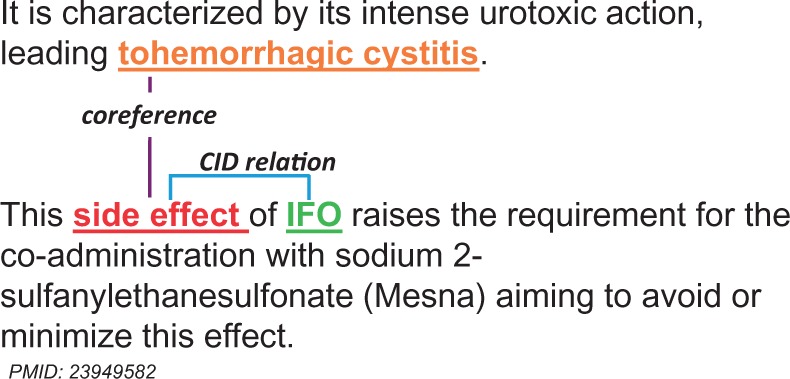
An example of the coreference between chemical entities. Two sequential sentences are extracted from PubMed abstract PMID: 23949582.

Formally, the coreference consists of two linguistic expressions—antecedent and anaphor (19). Figure 4 is an example of the coreference, in which the anaphor ‘side effect’ is the expression whose interpretation depends on that of the other expression, and the antecedent ‘tohemorrhagic cystitis’ is the linguistic expression on which an anaphor ‘side effect’ depends on.

The traditional coreference resolution task was normally to discover the antecedents for each anaphor in a document. From the perspective of this study, it was not necessary to always make clear which is the antecedent or anaphor. Our system considered both antecedents and anaphors as mentions of entities, and strived to recognize as many mentions of an entity as possible.

Studies on the coreference resolution in the general English domain date back to 1960s and 1970s and often focus on person, location and organization. In biomedicine, because entity types to be resolved are atypical to general domains (i.e. protein, gene, disease, chemical, etc.), coreference researches in this domain have received comparatively less attentions (19). Previous approaches had applied several methods, ranging from heuristics-based (20, 21) to machine learning (22, 23).

**Figure 5.**
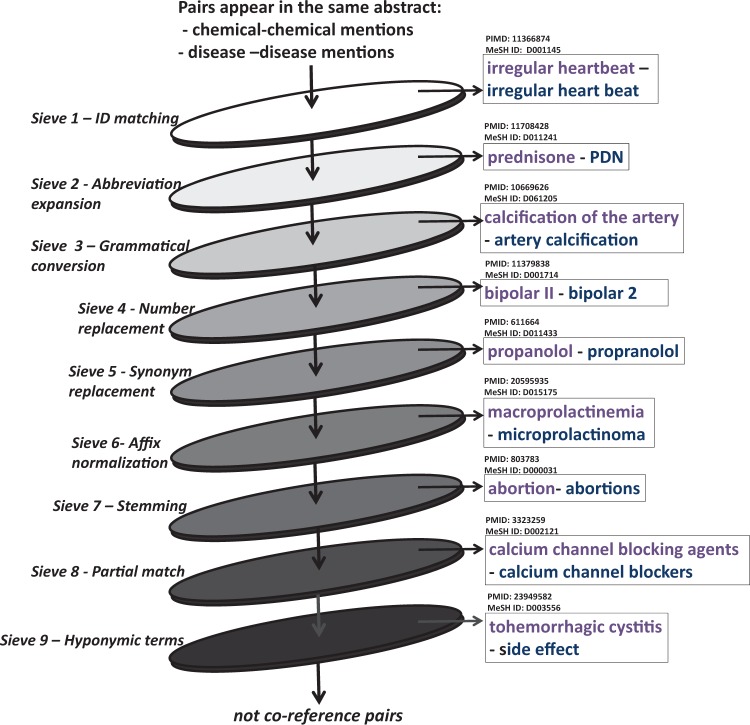
Coreference resolution using nine-pass sieve. Examples is pairs were kept by sieves.

In this regard, our proposed system employed the coreference module that was based on a multi-pass sieve model (21). It has been evaluated as a simple yet effective mean for disorder mention normalization (21). We first processed each abstract by noun phrase (NP) chunking (using Genia tagger; http://www.nactem.ac.uk/GENIA/tagger/) and then created a set of NPs pairs for each abstract. These pairs of NPs were then passed through the sieves. Those that were kept by any sieve were considered as coreferent pairs, those that were not kept in each sieve were passed through the next sieve to the end. There were nine sieves used, each corresponded to a set of rules. Figure 5 is an illustration of the sieve-based coreference resolution module with example pairs that were kept by each sieve.

Sieve 1—ID matching: Two chemical or disease mentions that have the same MeSH ID are coreferent. This sieve used information from the previous NEN step. For example as ‘irregular heartbeat’ and ‘irregular heart beat’ were both normalized to MeSH ID: D001145, and were thus considered coreferent.

Sieve 2—Abbreviation expansion: In this sieve we used the BioText Abbreviation recognition software (http://biotext.berkeley.edu/software.html) (24) to identify abbreviations and their full forms (e.g. full form of ‘PND’ in the abstract PMID:11708428 is ‘prednisone’). We then checked the MeSH ID of the full form and applied it to the abbreviation in order to unify mentions.

Sieve 3—Grammatical conversion: Similar forms of an entity mention were automatically generated by changing grammatical elements in mentions, including subjects, objects and prepositions, etc. The ID match criterion was then checked. New forms were obtained by applying rules proposed by D’Souza and Ng (21), which includes: (i) replacing the preposition in the name with other prepositions, (ii) dropping the preposition from the name and swapping the substring surrounding it, (iii) bringing the last token to the front, inserting a preposition as the second token, and shifting the remaining tokens to right by two and (iv) moving the first token to the end, inserting a preposition as the second to last token, and shifting the remaining tokens to the left by two. Examples include ‘calcification of the artery’ and ‘artery calcification’, ‘mental status alteration’ and ‘alteration in mental status’.

Sieve 4—Number replacement: Similar forms of a mention were generated by replacing numbers with other forms and the ID match criterion was checked. In this regard, we considered the numeral, roman numeral, cardinal and multiplicative forms of a number for generating new mention forms, i.e. ‘two’ can be converted to ‘2’, ‘ii’ and ‘double’.

Sieve 5—Synonym replacement: The ID match criterion for synonyms of mentions was checked. This sieve used a synonym dictionary constructed from the MeSH, which contains 780 982 entries. Examples include ‘propanolol’ and ‘propranolol’.

Sieve 6—Affix normalization: New forms of a mention were generated by changing affixes (including prefixes and suffixes) and then the ID match criterion was checked. For examples, ‘macroprolactinemia’ and ‘microprolactinoma’ (PMID:20595935), ‘nephrotoxicity’ and ‘nephrotoxic’ (PMID:19642243) are coreferent.

Sieve 7—Stemming: Entity mentions are stemmed using the Porter stemmer (http://tartarus.org/martin/Porter Stemmer/), and then the ID match criterion was checked. Examples include ‘abortion’ and ‘abortions’.

Sieve 8—Partial match: This sieve used the output information from the abbreviation expansion sieve and applied the criterion for partial matching proposed by D’Souza and Ng (21). It is said that ‘a mention can be partially matched with another mention for which it shares the most tokens’. To give an example, ‘calcium channel blocking agents’ and ‘calcium channel blockers’ in abstract PMID:3323259 were marked as coreference.

Sieve 9—Hyponymic terms: We created two dictionaries for chemicals and diseases including hyponymic nouns that often referred to chemicals/diseases. For example, chemical hyponymic dictionary includes ‘drug’, ‘dose’, etc.; disease hyponymic dictionary includes ‘disease’, ‘case’, ‘infection’, ‘side effect’, etc. In this sieve, NER information was used to find chemical and disease entities, and if there was any term in dictionary within its context window of two sentences before-/after-ward, we could determine a coreference.

#### SVM-based intra-sentence relation extraction

Our work was based on the know-how that if a NP and an entity are coreferent, the NP can be considered as an entity of that type. The intra-sentence relation extraction module received sentences that contain a disease—chemical pair as input and classified whether this pair had the CID relation or not.

The example in Figure 4 (section Coreference resolution) also shows how to combine the coreference resolution module and the intra-sentence relation extraction module for handling inter-sentence relations. The strategy is that if the intra-sentence relation extraction module can recognize the relation between ‘side effect’ and ‘IFO’, we can also determine the relation between ‘tohemorrhagic cystitis’ and ‘IFO’ because ‘tohemorrhagic cystitis’ and ‘side effect’ are coreferent.

The intra-sentence relation extraction module was based on a SVM (25)—one of the most popular machine learning methods that has been successfully applied for biomedical relation extraction (26, 27). We used the Liblinear tool (http://www.csie.ntu.edu.tw/∼cjlin/liblinear/) to train a supervision binary SVM classifier (L2- regularized and L1-loss) on the CDR track training/development data set and our SilverCID corpus. In this study, we observed that the complexities of CID relations (several structural forms, abundance-related vocabulary sets, difficulty to determine the distance between the two entities, etc.) are very similar to the event extraction problem. As a consequence, the feature set that was specially constructed for event extraction might work better than that commonly used for normal relation extraction [they were words, entity types, mention levels, overlap, dependency, parse tree and dictionary (28–30)]. Following a report of high performance in event extraction (31), we decided to use a large-scale feature set including four types of features: Token features, neighboring token features, token features n-gram, pair features n-gram and shortest features path, the feature’s details are shown in Table 2.

**Table 2.** Large-scale feature set used in the intra-sentence relation extraction module

Feature types	Description	Features	Provided information
Token features	Token itself	Token orthography (capitalization, first letter of sentence, number, etc.) Base form of token N-grams (*n* = 1–4) of token Part-of-speech tagging	Information about the current token
Neighboring token features	Extracts all 2-step dependency paths from the target token, which then were used to extract n-grams	Features extracted by the token feature function for each token Token and dependency n-grams (*n* *=* 2–4) Token n-grams (*n* = 2; 3) Dependency n-grams (*n* = 2)	Information about the surrounding context of the current token
Token n-gram features	Extract token n-grams (*n*=1–4) within a window of three tokens before and three tokens after the target token	N-grams of word	Information about phrase which contain current token
Pair n-gram features	Extracts word n-grams (*n*=1–4) within a window from three tokens before the first tokens to three tokens after the last token in target chemical-disease pair.	Dependency n-grams (*n* = 2) Token n-grams (*n* = 2, 3) N-grams (*n* = 2–4) of dependencies and tokens	Information about function of current token in the dependency tree
Shortest path features	Shortest dependency paths between two words (in which, each word belongs to a disease or chemical entity)	Length of path Word n-grams (*n* = 2– 4) Dependency n-grams (*n* = 2–4) Consecutive word n-grams (*n* = 1–3) representing governor-dependent relationships Edge walks (word-dependency-word) and their sub-structures Vertex walks (dependency-word-dependency) and their sub-structures	Information about relation between current token and other tokens in sentence using dependency tree and function of each token in this path

## Experimental results

For evaluation, disease entities and CID relations that had been predicted by our proposed model were compared to the gold standard annotated CDR testing data set using standard metrics: precision (P, indicating the percentage of predicted positives that are true instances), recall (R, indicating the percentage of true positive instances that the system has retrieved) and F1 (the harmonic means of R and P).

BioCreative V also evaluates the running time of participating systems based on response time via teams’ respective web services.

### DNER results

The experimental results of the DNER phase on the CDR track testing data set are shown in Table 3. Note that only disease entities were evaluated.

**Table 3.** DNER results

		**P (%)**	**R (%)**	**F (%)**
BioCreative benchmarks^a^	Dictionary look-up	42.71	67.46	52.30
	DNorm	81.15	80.13	80.64
	Average results	78.99	74.81	76.03
	Ranked no. 1 result	89.63	83.50	86.46
**Our system in BioCreative V **(33)^a^		**73.20**	**79.98**	**76.44**
**Our improved system**^b^		**79.90**	**85.16**	**82.44**
NER-NEN pipeline		78.26	83.17	80.64

a Provided by the BioCreative 2015 organizer (33).

b The silverCID corpus included in training the NER module.

We compared our results with the benchmarks provided by the BioCreative organizer, including:

- The straightforward dictionary look-up method that relied on disease names from the CTD database.
- Retrained models using the out-of-box DNorm (16), which was a competitive system that achieved the highest performance in a previous disease challenge. DNorm combined an approach that was based on conditional random fields (CRFs) and rich features for NER with a pair wise learning to rank for NEN.
- BioCreative DNER average results: Average results of the best run of 16 teams participating in the DNER task.
- BioCreative DNER no. 1 ranked team results: Results from the team that was ranked no. 1 (in term of F1) in the DNER task (32). This system used a linear chain CRF with rich features for NER, they used three lexicons resources to generate CRF dictionary features and multiple post processing steps to optimize the results. In the NEN step, they used a dictionary-lookup method that was based on the collection of MEDI, NCBI disease corpus and the CDR task data set.

In this article, we improved our system (33) that had participated in the BioCreative DNER task by adding the silverCID corpus in the NER averaged perceptron training set. Table 3 also shows how useful the silverCID was in boosting the performance of our proposed model.

In the BioCreative V evaluation, our system performed far beyond the dictionary look up method, but worse than DNorm that was considered as a very strong benchmark (note that there were only seven participating teams that achieved performances better than DNorm). Using the silverCID corpus for training NER model boosted the performance by 6% of F1 and became better than the DNorm’s result.

To demonstrate the benefit of the joint decoding model, we also built a baseline system that was based on the traditional pipeline model: NER was employed first and its result was then used for NEN. In this manner, the NER and NEN modules were totally similar with them in our joint decoding model. The results showed that joint decoding model boosted the performance by 1.8% of the F1 score.

Following the results reported by BioCreative (8), the average response time in the DNER task was 5.6 s and our system was among participating systems that had smallest response time (276 ms, ranked no. 2).

### CID results

Table 4 shows the results of our system on the CID task. It serves two purposes, i.e. firstly for comparing our results with the BioCreative benchmark results, and secondly for evaluating the contribution of the coreference resolution approach and the silver-CID corpus as well as finding the best combination of them.

**Table 4.** CID relation extraction results

		P (%)	R (%)	F (%)
BioCreative benchmarks^a^	Co-occurrence^a^	16.43	76.45	27.05
	Average result^a^	47.09	42.61	43.37
	Rank no. 1 result^a^	55.67	58.44	57.03
**Our system in BioCreative V** (33)^a^		**53.41**	**49.91**	**51.60**
**(SVM+ CR MPS)**				
**Our improved system**		**57.63**	**60.23**	**58.90**
**(SVM+ CR MPS+ silverCID corpus)**				
SVM		44.73	50.56	47.47
SVM+ silverCID corpus		51.42	52.81	52.11
SVM+ CR EMC		47.64	50.28	48.93

CR, coreference resolution; MPS, multi-pass sieve; EMC, EM clustering.

a Results provided by the BioCreative 2015 organizer (33). SVM: SVM intra-sentence relation extraction. Bold values are performance measures of our two models on the Test set, not using cross-validation evaluation.

We compared our results with the benchmarks provided by the BioCreative organizer, including:

- The co-occurrence baseline method with two variants: abstract-level and sentence-level.
- BioCreative CID average results: Average results of the best run of 18 teams participating in the CID task.
- BioCreative CID no. 1 ranked team results: Results from the team that was ranked no. 1 (in term of F1) in the CID task (34). This system combines two SVM classifiers trained on sentence- and document-level, its novel aspect is at using rich features coming from CID relations in other biomedical resources.

The configuration of our system that participated in the CID task was the pipeline of a multi-pass sieve coreference resolution module and a SVM intra-sentences relation extraction module. It achieved the F1 score of 51.60%. This was much better than that of the co-occurrence benchmark method. Further, the improved system that used the SilverCID corpus for training SVM module boosted the performance by 7.3% of F1. It can be noted that this result is better than that of the highest ranked system in the CID task. The contributions of the coreference resolution and silverCID corpus were evaluated by comparing results of the SVM-based intra-sentence relation extraction module with and without adding the coreference resolution module/silverCID corpus. A comparative evaluation showed that the original SVM approach (only trained on the CDR training and development set) achieved F1 of 47.47%, whilst adding the SilverCID corpus boosted F1 by 4.64% (51.60%) and, further, adding the multi-pass sieve coreference resolution module boosted F1 by 4.13% more (58.90%).

We also made a comparison between our heuristic-based multi-pass sieve method and another state-of-the-art machine learning-based method for coreference resolution. In this regard, we re-implemented a method proposed by Ng (22), which was an expectation maximization (EM) clustering coreference approach. This system also used the SVM-based intra-sentence relation extraction model that had been trained on the CDR training and development set. The results demonstrated the strength of our multi-pass sieves method. We achieved 53.41% in precision (5.77% better than that of the EM clustering-based), 49.91% in recall (0.37% worse) and 51.60% in F1 (2.67% better).

The feature set that was used in the SVM model contains 332 570 features. It is clearly a non-trivial large feature space to compute. In our experiments, the SVM model took more than an hour for training. According to the results reported by BioCreative (8), the average response time in the CID task was 9.3 s. Our system response time was 8.993 s.

## Discussion

Our work makes available to others the SilverCID corpus which was built automatically, in addition to the extant resources, i.e. the CDR track data set (section BioCreative CDR track data set) and the CTD-Pfizer collaboration data set (11) which have resulted from manual curation. A further novel aspect that makes the SilverCID corpus different from other sources is that it is a sentence-level corpus which covered about 60% of CID relations in CTD database.

Traditionally, NER and NEN were treated as two separate tasks, in which, NEN took the output of NER as its input. Following several studies, e.g. Liu *et al.* (35), we begin to understand the limitations of this pipeline approach, i.e. the errors propagate from NER to NEN and there is no feedback from NEN to NER. Khalid *et al.* (36) also demonstrated that most NEN errors were caused by recognition errors. The joint decoding model is expected to overcome these disadvantages of such a traditional pipeline model. Table 3 shows that joint decoding model boosted performance by 1.8% in term of the F1 score. Joint decoding outperformed the pipeline model in the cases of long entities that belongs to MeSH, such as ‘combined oral contraceptives’ (MeSH:68003277) and ‘angiotensin-converting enzyme inhibitors’ (MeSH:D000806).

In the DNER phase, the NEN back-off model could take advantage of both labeled CDR data set and the extremely large unlabeled PubMed data. The SSI model calculated the correlation matrix between tokens, it worked better than Skip-gram in cases that tokens appeared in training data or MeSH (e.g. SSI links ‘arrhythmias’ to MeSH:D001145 (Arrhythmias, Cardiac), ‘peripheral neurotoxicity’ to MeSH:D010523 (Peripheral Nervous System Diseases). The skip-gram model calculated similarity between tokens by taking advantage of the large unlabeled PubMed data, and helped improve the system recall (e.g. Skip-gram linked ‘disordered gastrointestinal motility’ to MeSH:D005767 (Gastrointestinal Diseases), ‘hyperplastic marrow’ to MeSH:D001855 (Bone Marrow Diseases), which were false negatives by the SSI model).

In the CID phase, we compared some true positive results of three other systems which are listed in Table 5. The comparing systems include: (i) SVM model that was only trained on the CDR training and development data, (ii) pipeline model of the SVM module and the multi-pass sieve coreference resolution module and (iii) the same model as in (ii), but with the Silver-CID corpus used for training the SVM module. The disagreements between these three systems, which are shown in Table 5, clarify contribution of the method and data set used in our model (see Supplementary 1 for the sample texts).

**Table 5.** Analysis of the contribution of methods and resources used in our proposed system for capturing CID relationships

	CID relation (chemical-disease)	PMID	**Type of relation**		SVM	SVM+CR	SVM+SC	SVM+CR+SC
			Intra-	Inter-				
1	Maleate (C030272)—nephrotoxicity (D007674)	25119790	✓		✓	✓	✓	✓
2	Quinacrine hydrochloride (D011796)—atrial thrombosis (D003328)	6517710	✓		✓	✓		
3	Metolachlor (C051786) -liver cancer (D008113)	26033014	✓				✓	✓
4	Galantamine (D005702) – headaches (D006261)	17069550	✓					
5	Methoxamine (D008729)- headache (D006261)	11135381		✓		✓		✓
6	Gemfibrozil (D015248)—myositis (D009220)	1615846		✓				✓
7	Oxidized and reduced glutathione (D019803) —reperfusion injury (D015427)	1943082		✓		✓		
8	Metolachlor (C051786)- follicular cell lymphoma (D008224)	26033014		✓				

SVM, SVM intra-sentence relation extraction; CR, multi-pass sieve coreference resolution; SC, silverCID corpus; Intra-, Intra-sentence CID relation; Inter-, Inter-sentence CID relation; ✓, chemical-disease pair is classified as CID relation correctly. See supplementary 1 for the sample texts.

First, Table 5 shows that the SVM-based intra-sentence relation extraction model played the central core of our system. It worked well in the cases of intra-sentence CID relations (e.g. examples 1 and 2). As a result, if SVM failed on an intra-sentence relation, adding the multi-pass sieve coreference resolution module was not helpful (e.g. examples 3 and 4).

Since the SilverCID corpus enriched the training data for SVM, using it might help to find more relations than only the SVM model did (e.g. example 3). It, however, also might bring some noises leading to the small adverse effects for the system, i.e. adding the silverCID could lead to some missing results (e.g. example 2).

It is certain that SVM only based model, even trained on the SilverCID corpus or not, could not catch the inter-sentence relations (e.g. examples 5–8). Therefore, the coreference resolution was completely necessary for handling the inter-sentence relations (e.g. examples 5 and 7). Similar to intra-sentence relation cases, adding the silverCID corpus might help (e.g. example^6^) or reject very small amount true positives classified by the SVM + coreference model (e.g. example 7). 

Furthermore, there were still many cases on that the systems as a whole failed (e.g. examples 4 and 8).

Table 6 shows examples of where our system disagreed with the annotation standard (see Supplementary 2 for example texts). There were two types of errors: wrong results (FP) and missing results (FN). Since entities which participated in the CID relations were expressed using their MeSH ID and the evaluation was made at the abstract-level, it was very hard to clarify the cause of errors within the whole system. Our comments for these cases were empirical, based on heuristic surveying the system output.

**Table 6.** Sources of errors by our system on the CDR test set

	CID relation (chemical-disease)	PMID	**Type of error**		Cause of error
			FP	FN	
1	Corticosteroid (D000305)–systemic sclerosis (D012595)	22836123		✓	Complex inter-sentence structure
2	Cyclophosphamide (D003520)–edema (D004487)	23666265		✓	Complex inter-sentence structure
3	Chlorhexidine diphosphanilate (C048279)–pain (D010146)	2383364		✓	Noise from silverCID corpus
4	Theophylline (D013806)–tremors (D014202)	3074291		✓	Error from NER
5	Scopolamine (D012601)–retention deficit (D012153)	3088653	✓		Error from NER
6	Clopidogrel (C055162)–acute hepatitis (D017114)	23846525	✓		Error from NER
7	Isoproterenol (D007545)–heart hypertrophy (D006984)	2974281	✓		Error from NEN
8	Nicotine (D009538)–anxiety (D001008)	15991002	✓		Noise from silverCID corpus
9	Oxitropium bromide (C017590)–nausea (D009325)	3074291	✓		Error from SVM model
10	Gamma-vinyl-GABA (D020888)–status epilepticus (D013226)	3708328	✓		Error from coreference resolution module

Intra-, Intra-sentence CID relation; Inter-, Inter-sentence CID relation; FP, false positive; FN, false negative. See supplementary 2 for the sample texts.

In Table 6, some errors were caused by the previous DNER phase: in example 4, NER module did not recognize ‘theophylline’ as chemical; in example 5, FP result for the ‘retention deficit’ of NER module leaded to the FP error of the whole system; in example 6, NER determined the wrong boundary of the entity ‘acute hepatitis’ and in example 7, NEN module matched ‘heart hypertrophy’ to the wrong MeSH ID (it should be ‘D006332’).

Inter-sentence relations often have very complex structures. Two entities involving such relations might belong to two sentences that were not adjacent. In some worst cases, one entity was even hidden, which caused many FN errors (e.g. examples 1 and 2).

The SVM module depended on the training data set, thus, it might lead to several limitations of finding new relations (which were not similar with those in the training set). Example 9 in Table 6 demonstrates this type of limitation.

Coreference resolution was not a trivial problem, it had several types of errors by itself. The FP error in example 10 seems to be caused by the coreference resolution module, i.e. linking the term ‘dose’ to the wrong entity.

Regarding errors caused by using the silverCID corpus, we noted that this corpus might bring much valuable information, but it also might bring some noise, leaded to FN errors (e.g. example 3) and FP errors (e.g. example 8). Such two errors would disappear if we had removed the silverCID corpus from our system.

## Conclusions

In this research, we have presented a systematic study of our approach to the BioCreative V CDR task. Improvements on the original system include: (i) a joint decoding approach for NER and NEN based on several state-of-the-art machine learning methods for the DNER sub-task and (ii) improvements of a SVM-based model for the CID sub-task by using a large-scale feature set, silverCID corpus and crucially, a multi-pass sieve coreference resolution module. Our best performance achieved an F1 of 81.93 for DNER while that of the DNorm, the state-of-the-art DNER system based on SSI, was 80.64%. The best performance for CID of our improved system had F1 of 58.90, comparable to 57.03% of the highest ranked system in the CID task.

Based on the CTD database, we built a silver standard data set (called ‘silverCID’ corpus), including 51 719 sentences that contained CID relations with silver annotations for NER, NEN and CID relations. The use of the SilverCID corpus would not have been allowed under the original rules of the task because it was unknown which subset of the database was used in the test evaluation. In our comparison, this SilverCID corpus proved its effectiveness when boosting our system performance by 7.3% in term of the F1 score (note that we checked to make sure that there were no overlap between CTD-silver set and the test set).

Several comparisons were made to compare our results with those of other systems and to analyze the system errors. The evidences pointed towards complementarities between the NER-NEN joint decoding model, the SVM model, the SilverCID corpus and the coreference resolution module. The empirical results also demonstrated the advantage of using multi-pass sieve coreference resolution to handle inter-sentence relations.

One limitation of our system is that DNER was the initial step of CID, thus, DNER results greatly influenced the CID results. Therefore, the comparison hereby required further validations because we used NER and NEN information provided by our DNER phase while other systems used theirs.

Our proposed system is extensible in several ways. Improving the coreference resolution module is obviously the first possible follow-up. Although the coreference resolution module plays a central role in extracting inter-relations, at this time, it only boosted the performance by 4.13% in terms of F1. One potential suggestion is to use the SilverCID corpus for training a multi-pass sieve coreference module as the more results the coreference resolution module can find out, the more inter-relations can be found. The second possible follow-up to improve our system may come from several useful biomedical resources that we did not utilize. According to the report of the best team in the DNER sub-task of BioCreative V (32), we know that they exploited many databases such as CTD, MEDI (37), SIDER (38), etc. to extract various useful knowledgebase features for their machine learning based participating system or to be as a dictionary for matching. The third can be application of several post-processing steps, such as abbreviation resolution and consistency improvement, which was applied by the best team in the DNER sub-task of the BioCreative V and demonstrated its effectiveness (31).

## Supplementary Material

Supplementary Data
