# Supplementary material for: Sieve-based coreference resolution enhances semi-supervised learning model for chemical-induced disease relation extraction
Source: Database (Oxford). 2016 Jul 26;2016:baw102. doi: 10.1093/database/baw102 (PMC4962668; doi:10.1093/database/baw102)
Supplement: Supplementary Data [file supp_2016_baw102_index.html]

Supplementary Data 

# Sieve-based coreference resolution enhances semi-supervised learning model for chemical-induced disease relation extraction

## Supplementary Data

files

- Supplementary Data - zip file
