# Supplementary material for: Sieve-based coreference resolution enhances semi-supervised learning model for chemical-induced disease relation extraction
Source: Database (Oxford). 2016 Jul 26;2016:baw102. doi: 10.1093/database/baw102 (PMC4962668; doi:10.1093/database/baw102)
Supplement: Supplementary Data [file supp_baw102_suppl_data.zip › Supplementary 1.doc]

**Table 5**. Analysis of the contribution of methods and resources used in the proposed system for capturing CID relationships

|  | **CID relation (chemical-disease)** | **PMID** | **Type of relation** | | **SVM** | **SVM**  **+CR** | **SVM**  **+SC** | **SVM**  **+CR**  **+SC** |
| --- | --- | --- | --- | --- | --- | --- | --- | --- |
|  |  |  | **Intra-** | **Inter-** |  |  |  |  |
| 1 | maleate (C030272) - nephrotoxicity (D007674) | 25119790 | ✓ |  | ✓ | ✓ | ✓ | ✓ |
| 2 | quinacrine hydrochloride (D011796) - atrial thrombosis (D003328) | 6517710 | ✓ |  | ✓ | ✓ |  |  |
| 3 | metolachlor (C051786) -liver cancer (D008113) | 26033014 | ✓ |  |  |  | ✓ | ✓ |
| 4 | galantamine (D005702) – headaches (D006261) | 17069550 | ✓ |  |  |  |  |  |
| 5 | methoxamine (D008729)- headache (D006261) | 11135381 |  | ✓ |  | ✓ |  | ✓ |
| 6 | gemfibrozil (D015248) - myositis (D009220) | 1615846 |  | ✓ |  |  |  | ✓ |
| 7 | oxidized and reduced glutathione (D019803) - reperfusion injury (D015427) | 1943082 |  | ✓ |  | ✓ |  |  |
| 8 | metolachlor (C051786)- follicular cell lymphoma (D008224) | 26033014 |  | ✓ |  |  |  |  |

SVM: SVM intra-sentence relation extraction. CR: Multi-pass sieve coreference resolution. SC: silverCID corpus. Intra-: Intra-sentence CID relation. Inter-: Inter-sentence CID relation. ✓: Chemical-disease pair is classified as CID relation correctly. See supplementary 1 for the sample texts.

Chemical: Participated Chemical terms

Disease: Participated Disease terms

1. **PMID 25119790**

**Intra-sentence relation: maleate (C030272) - nephrotoxicity (D007674)**

… Curcumin prevents maleate-induced nephrotoxicity: relation to hemodynamic alterations, oxidative stress, mitochondrial oxygen consumption and activity of respiratory complex I…

2. **PMID 6517710**

**Intra-sentence relation: quinacrine hydrochloride (D011796) - atrial thrombosis (D003328)**
… Atrial thrombosis involving the heart of F-344 rats ingesting quinacrine hydrochloride…

3. **PMID 26033014**

**Intra-sentence relation: metolachlor (C051786) -liver cancer (D008113)**

… This suggestion of an association between metolachlor and liver cancer among pesticide applicators is a novel finding and echoes observation of increased liver neoplasms in some animal studies…

4. **PMID 17069550**

**Intra-sentence relation: galantamine (D005702) – headaches (D006261)**

… Overall, galantamine was well-tolerated, with no significant adverse effects apart from headaches in one patient…

5. **PMID 11135381**

**Inter-sentence relation: methoxamine (D008729)- headache (D006261)**

TITLE:

Effect of methoxamine on maximum urethral pressure in women with genuine stress incontinence: a placebo-controlled, double-blind crossover study.

ABSTRACT:

The aim of the study was to evaluate the potential role for a selective alpha1-adrenoceptor agonist in the treatment of urinary stress incontinence. A randomised, double-blind, placebo-controlled, crossover study design was employed. Half log incremental doses of intravenous methoxamine or placebo (saline) were administered to a group of women with genuine stress incontinence while measuring maximum urethral pressure (MUP), blood pressure, heart rate, and symptomatic side effects. Methoxamine evoked non-significant increases in MUP and diastolic blood pressure but caused a significant rise in systolic blood pressure and significant fall in heart rate at maximum dosage. Systemic side effects including piloerection, headache, and cold extremities were experienced in all subjects. The results indicate that the clinical usefulness of direct, peripherally acting sub-type-selective alpha1-adrenoceptor agonists in the medical treatment of stress incontinence may be limited by associated piloerection and cardiovascular side effects.

6. **PMID 1615846**

**Inter-sentence relation: gemfibrozil (D015248) - myositis (D009220)**

TITLE:

Gemfibrozil-lovastatin therapy for primary hyperlipoproteinemias.

ABSTRACT:

The specific aim of this retrospective, observational study was to assess safety and efficacy of long-term (21 months/patient), open-label, gemfibrozil-lovastatin treatment in 80 patients with primary mixed hyperlipidemia (68% of whom had atherosclerotic vascular disease). Because ideal lipid targets were not reached (low-density lipoprotein (LDL) cholesterol less than 130 mg/dl, high-density lipoprotein (HDL) cholesterol greater than 35 mg/dl, or total cholesterol/HDL cholesterol less than 4.5 mg/dl) with diet plus a single drug, gemfibrozil (1.2 g/day)-lovastatin (primarily 20 or 40 mg) treatment was given. Follow-up visits were scheduled with 2-drug therapy every 6 to 8 weeks, an average of 10.3 visits per patient, with 741 batteries of 6 liver function tests and 714 creatine phosphokinase levels measured. Only 1 of the 4,446 liver function tests (0.02%), a gamma glutamyl transferase, was greater than or equal to 3 times the upper normal limit. Of the 714 creatine phosphokinase levels, 9% were high; only 1 (0.1%) was greater than or equal to 3 times the upper normal limit. With 2-drug therapy, mean total cholesterol decreased 22% from 255 to 200 mg/dl, triglyceride levels decreased 35% from 236 to 154 mg/dl, LDL cholesterol decreased 26% from 176 to 131 mg/dl, and the total cholesterol/HDL cholesterol ratio decreased 24% from 7.1 to 5.4, all p less than or equal to 0.0001. Myositis, attributable to the drug combination and symptomatic enough to discontinue it, occurred in 3% of patients, and in 1% with concurrent high creatine phosphokinase (769 U/liter); no patients had rhabdomyolysis or myoglobinuria.(ABSTRACT TRUNCATED AT 250 WORDS)

7. **PMID 1943082**

**Inter-sentence relation: oxidized and reduced glutathione (D019803) - reperfusion injury (D015427)**

TITLE:

Hepatocellular oxidant stress following intestinal ischemia-reperfusion injury.

ABSTRACT:

Reperfusion of ischemic intestine results in acute liver dysfunction characterized by hepatocellular enzyme release into plasma, reduction in bile flow rate, and neutrophil sequestration within the liver. The pathophysiology underlying this acute hepatic injury is unknown. This study was undertaken to determine whether oxidants are associated with the hepatic injury and to determine the relative value of several indirect methods of assessing oxidant exposure in vivo. Rats were subjected to a standardized intestinal ischemia-reperfusion injury. Hepatic tissue was assayed for lipid peroxidation products and oxidized and reduced glutathione. There was no change in hepatic tissue total glutathione following intestinal ischemia-reperfusion injury…

8. **PMID 26033014**

**Inter-sentence relation: metolachlor (C051786)- follicular cell lymphoma (D008224)**

TITLE:

Cancer incidence and metolachlor use in the Agricultural Health Study: An update.

ABSTRACT:

Metolachlor, a widely used herbicide, is classified as a Group C carcinogen by the U.S. Environmental Protection Agency based on increased liver neoplasms in female rats. Epidemiologic studies of the health effects of metolachlor have been limited. The Agricultural Health Study (AHS) is a prospective cohort study including licensed private and commercial pesticide applicators in Iowa and North Carolina enrolled 1993-1997. We evaluated cancer incidence through 2010/2011 (NC/IA) for 49,616 applicators, 53% of whom reported ever using metolachlor. We used Poisson regression to evaluate relations between two metrics of metolachlor use (lifetime days, intensity-weighted lifetime days) and cancer incidence. We saw no association between metolachlor use and incidence of all cancers combined (n = 5,701 with a 5-year lag) or most site-specific cancers. For liver cancer, in analyses restricted to exposed workers, elevations observed at higher categories of use were not statistically significant. However, trends for both lifetime and intensity-weighted lifetime days of metolachor use were positive and statistically significant with an unexposed reference group. A similar pattern was observed for follicular cell lymphoma, but no other lymphoma subtypes. An earlier suggestion of increased lung cancer risk at high levels of metolachlor use in this cohort was not confirmed in this update. This suggestion of an association between metolachlor and liver cancer among pesticide applicators is a novel finding and echoes observation of increased liver neoplasms in some animal studies. However, our findings for both liver cancer and follicular cell lymphoma warrant follow-up to better differentiate effects of metolachlor use from other factors.
