# Supplementary material for: Sieve-based coreference resolution enhances semi-supervised learning model for chemical-induced disease relation extraction
Source: Database (Oxford). 2016 Jul 26;2016:baw102. doi: 10.1093/database/baw102 (PMC4962668; doi:10.1093/database/baw102)
Supplement: Supplementary Data [file supp_baw102_suppl_data.zip › Supplementary 2.doc]

**Table 6**.  Sources of errors by our system system on the CDR test set

|  | **CID relation (chemical-disease)** | **PMID** | **Type of error** | | **Cause of error** |
| --- | --- | --- | --- | --- | --- |
|  |  |  | **FP** | **FN** |  |
| 1 | corticosteroid (D000305) - systemic sclerosis (D012595) | 22836123 |  | ✓ | Complex inter-sentence structure |
| 2 | cyclophosphamide (D003520) - edema (D004487) | 23666265 |  | ✓ | Complex inter-sentence structure |
| 3 | chlorhexidine diphosphanilate (C048279) - pain (D010146) | 2383364 |  | ✓ | Noise from silverCID corpus |
| 4 | theophylline (D013806) - tremors (D014202) | 3074291 |  | ✓ | Error from NER |
| 5 | scopolamine (D012601) - retention deficit (D012153) | 3088653 | ✓ |  | Error from NER |
| 6 | clopidogrel (C055162) - acute hepatitis (D017114) | 23846525 | ✓ |  | Error from NER |
| 7 | isoproterenol (D007545) - heart hypertrophy (D006984) | 2974281 | ✓ |  | Error from NEN |
| 8 | nicotine (D009538) - anxiety (D001008) | 15991002 | ✓ |  | Noise from silverCID corpus |
| 9 | oxitropium bromide (C017590) - nausea (D009325) | 3074291 | ✓ |  | Error from SVM model |
| 10 | gamma-vinyl-GABA (D020888) - status epilepticus (D013226) | 3708328 | ✓ |  | Error from coreference resolution module |

Intra-: Intra-sentence CID relation. Inter-: Inter-sentence CID relation. FP: False positive. FN: False negative. See supplementary 2 for the sample texts.

Chemical: Participated Chemical terms

Disease: Participated Disease terms

1. **PMID 22836123**

**FN Relation: corticosteroid (D000305) - systemic sclerosis (D012595)**

TITLE:

Late-onset scleroderma renal crisis induced by tacrolimus and prednisolone: a case report.

ABSTRACT:

Scleroderma renal crisis (SRC) is a rare complication of systemic sclerosis (SSc) but can be severe enough to require temporary or permanent renal replacement therapy. Moderate to high dose corticosteroid use is recognized as a major risk factor for SRC. Furthermore, there have been reports of thrombotic microangiopathy precipitated by cyclosporine in patients with SSc. In this article, we report a patient with SRC induced by tacrolimus and corticosteroids. The aim of this work is to call attention to the risk of tacrolimus use in patients with SSc.

2. **PMID 23666265**

**FN Relation: cyclophosphamide (D003520) - edema (D004487)**

TITLE:

The function of P2X3 receptor and NK1 receptor antagonists on cyclophosphamide-induced cystitis in rats.

ABSTRACT:

PURPOSE: The purpose of the study is to explore the function of P2X3 and NK1 receptors antagonists on cyclophosphamide(CYP)-induced cystitis in rats. METHODS: Sixty female Sprague-Dawley (SD) rats were randomly divided into three groups. The rats in the control group were intraperitoneally (i.p.) injected with 0.9% saline (4 ml/kg); the rats in the model group were i.p. injected with CYP (150 mg/kg); and the rats in the intervention group were i.p. injected with CYP with subsequently perfusion of bladder with P2X3 and NK1 receptors' antagonists, Suramin and GR 82334. Spontaneous pain behaviors following the administration of CYP were observed. Urodynamic parameters, bladder pressure-volume curve, maximum voiding pressure (MVP), and maximum cystometric capacity (MCC), were recorded. Pathological changes in bladder tissue were observed. Immunofluorescence was used to detect the expression of P2X3 and NK1 receptors in bladder.

RESULTS: Cyclophosphamide treatment increased the spontaneous pain behaviors scores. The incidence of bladder instability during urine storage period of model group was significantly higher than intervention group (X(2) = 7.619, P = 0.007) and control group (X(2) = 13.755, P = 0.000). MCC in the model group was lower than the control and intervention groups (P < 0.01). Histological changes evident in model and intervention groups rats' bladder included edema, vasodilation, and infiltration of inflammatory cells. In model group, the expression of P2X3 receptor increased in urothelium and suburothelium, and NK1 receptor increased in suburothelium, while the expression of them in intervention group was lower.

CONCLUSIONS: In CYP-induced cystitis, the expression of P2X3 and NK1 receptors increased in urothelium and/or suburothelium. Perfusion of bladder with P2X3 and NK1 receptors antagonists ameliorated the bladder function.

3. **PMID 2383364**

**FN Relation: chlorhexidine diphosphanilate (C048279) - pain (D010146)**

TITLE:

Patient tolerance study of topical chlorhexidine diphosphanilate: a new topical agent for burns.

ABSTRACT:

Effective topical antimicrobial agents decrease infection and mortality in burn patients. Chlorhexidine phosphanilate (CHP), a new broad-spectrum antimicrobial agent, has been evaluated as a topical burn wound dressing in cream form, but preliminary clinical trials reported that it was painful upon application. This study compared various concentrations of CHPto determine if a tolerable concentration could be identified with retention of antimicrobial efficacy. Twenty-nine burnpatients, each with two similar burns which could be separately treated, were given pairs of treatments at successive 12-h intervals over a 3-day period. One burn site was treated with each of four different CHP concentrations, from 0.25 per cent to 2 per cent, their vehicle, and 1 per cent silver sulphadiazine (AgSD) cream, an antimicrobial agent frequently used for topical treatment of burn wounds. The other site was always treated with AgSD cream. There was a direct relationship between CHPconcentration and patients' ratings of pain on an analogue scale. The 0.25 per cent CHP cream was closest to AgSD in paintolerance; however, none of the treatments differed statistically from AgSD or from each other. In addition, ease of application of CHP creams was less satisfactory than that of AgSD. It was concluded that formulations at or below 0.5 per cent CHP may prove acceptable for wound care, but the vehicle system needs pharmaceutical improvement to render it more tolerable and easier to use.

4. **PMID 3074291**

**FN Relation: theophylline (D013806) - tremors (D014202)**

TITLE:

Comparison of the effect of oxitropium bromide and of slow-release theophylline on nocturnal asthma.

ABSTRACT:

The effects of a new inhaled antimuscarinic drug, oxitropium bromide, and of a slow-release theophylline preparation upon nocturnal asthma were compared in a placebo-controlled double-blind study. Two samples were studied: 12 patients receivedoxitropium at 600 micrograms (6 subjects) or at 400 micrograms t.i.d. (6 subjects) whereas 11 received theophylline at 300 mg b.i.d. Morning dipping, assessed by the fall in peak flow overnight, was significantly reduced in the periods when either active drug was taken, whereas no difference was noticed during the placebo administration. No significant difference was noticed between results obtained with either active drug, as well as with either dosage of oxitropium. No subject reported side effects of oxitropium, as compared to three subjects reporting nausea, vomiting and tremors after theophylline.Oxitropium proves to be a valuable alternative to theophylline in nocturnal asthma, since it is equally potent, safer and does not require the titration of dosage.

5. **PMID 3088653**

**FP Relation: scopolamine (D012601) - retention deficit (D012153)**

TITLE:

Reversal of scopolamine-induced amnesia of passive avoidance by pre- and post-training naloxone.

ABSTRACT:

In a series of five experiments, the modulating role of naloxone on a scopolamine-induced retention deficit in a passive avoidance paradigm was investigated in mice. Scopolamine, but not methyl scopolamine (1 and 3 mg/kg), induced an amnesia as measured by latency and duration parameters. Naloxone (0.3, 1, 3, and 10 mg/kg) injected prior to training attenuated the retention deficit with a peak of activity at 3 mg/kg. The effect of naloxone could be antagonized with morphine (1, 3, and 10 mg/kg), demonstrating the opioid specificity of the naloxone effect. Post-training administration of naloxone (3 mg/kg) as a single or as a split dose also attenuated the scopolamine-induced amnesia. Control experiments indicated that neither an increase in pain sensitivity (pre-training naloxone) nor an induced aversive state (post-training naloxone) appear to be responsible for the influence of naloxone on the scopolamine-induced retention deficit. These results extend previous findings implicating a cholinergic-opioid interaction in memory processes. A possible mechanism for this interaction involving the septo-hippocampal cholinergic pathway is discussed.

6. **PMID 23846525**

**FP Relation: clopidogrel (C055162) - acute hepatitis (D017114)**

TITLE:

Acute hepatitis associated with clopidogrel: a case report and review of the literature.

ABSTRACT:

Drug-induced hepatotoxicity is a common cause of acute hepatitis, and the recognition of the responsible drug may be difficult. We describe a case of clopidogrel-related acute hepatitis. The diagnosis is strongly suggested by an accurate medical history and liver biopsy. Reports about cases of hepatotoxicity due to clopidogrel are increasing in the last few years, after the increased use of this drug. In conclusion, we believe that physicians should carefully consider the risk of drug-induced hepatic injury when clopidogrel is prescribed.

7. **PMID 2974281**

**FP Relation: isoproterenol (D007545) - heart hypertrophy (D006984)**

TITLE:

Subacute effects of propranolol and B 24/76 on isoproterenol-induced rat heart hypertrophy in correlation with blood pressure.

ABSTRACT:

We compared the potential beta-receptor blocker, B 24/76 i.e. 1-(2,4-dichlorophenoxy)-3[2-3,4-dimethoxyphenyl)ethanolamino]-prop an-2-ol, which is characterized by beta 1-adrenoceptor blocking and beta 2-adrenoceptor stimulating properties with propranolol. The studies were performed using an experimental model of isoproterenol-induced heart hypertrophy in rats. A correlation of the blood pressure was neither found in the development nor in the attempt to suppress the development of heart hypertrophy with the two beta-receptor blockers. Both beta-blockers influenced the development of hypertrophy to a different, but not reproducible extent. It was possible to suppress the increased ornithine decarboxylase activity with both beta-blockers in hypertrophied hearts, but there was no effect on the heart mass. Neither propranolol nor B 24/76 could stop the changes in the characteristic myosin isoenzyme pattern of the hypertrophied rat heart. Thus, the investigations did not provide any evidence that the beta-receptor blockers propranolol and B 24/76 have the potency to prevent isoproterenol from producing heart hypertrophy.

8. **PMID 15991002**

**FP Relation: nicotine (D009538) - anxiety (D001008)**

TITLE:

Nicotine antagonizes caffeine- but not pentylenetetrazole-induced anxiogenic effect in mice.

ABSTRACT:

RATIONALE: Nicotine and caffeine are widely consumed licit psychoactive drugs worldwide. Epidemiological studies showed that they were generally used concurrently. Although some studies in experimental animals indicate clear pharmacological interactions between them, no studies have shown a specific interaction on anxiety responses. OBJECTIVES: The present study investigates the effects of nicotine on anxiety induced by caffeine and another anxiogenic drug, pentylenetetrazole, in mice. The elevated plus-maze (EPM) test was used to evaluate the effects of drugs on anxiety. METHODS: Adult male Swiss Webster mice (25-32 g) were given nicotine (0.05-0.25 mg/kg s.c.) or saline 10 min before caffeine (70 mg/kg i.p.) or pentylenetetrazole (15 and 30 mg/kg i.p.) injections. After 15 min, mice were evaluated for their open- and closed-arm time and entries on the EPM for a 10-min session. Locomotor activity was recorded for individual groups by using the same treatment protocol with the EPM test.

RESULTS: Nicotine (0.05-0.25 mg/kg) itself did not produce any significant effect in the EPM test, whereas caffeine (70 mg/kg) and pentylenetetrazole (30 mg/kg) produced an anxiogenic effect, apparent with decreases in open-arm time and entry. Nicotine (0.25 mg/kg) pretreatment blocked the caffeine- but not pentylenetetrazole-induced anxiety. Administration of each drug and their combinations did not produce any effect on locomotor activity.

CONCLUSIONS: Our results suggest that the antagonistic effect of nicotine on caffeine-induced anxiety is specific to caffeine, instead of a non-specific anxiolytic effect. Thus, it may extend the current findings on the interaction between nicotine and caffeine.

9. **PMID 3074291**

**FP Relation: oxitropium bromide (C017590) - nausea (D009325)**

TITLE:

Comparison of the effect of oxitropium bromide and of slow-release theophylline on nocturnal asthma.

ABSTRACT:

The effects of a new inhaled antimuscarinic drug, oxitropium bromide, and of a slow-release theophylline preparation upon nocturnal asthma were compared in a placebo-controlled double-blind study. Two samples were studied: 12 patients received oxitropium at 600 micrograms (6 subjects) or at 400 micrograms t.i.d. (6 subjects) whereas 11 received theophylline at 300 mg b.i.d. Morning dipping, assessed by the fall in peak flow overnight, was significantly reduced in the periods when either active drug was taken, whereas no difference was noticed during the placebo administration. No significant difference was noticed between results obtained with either active drug, as well as with either dosage of oxitropium. No subject reported side effects of oxitropium, as compared to three subjects reporting nausea, vomiting and tremors after theophylline.Oxitropium proves to be a valuable alternative to theophylline in nocturnal asthma, since it is equally potent, safer and does not require the titration of dosage.

10. **PMID 3708328**

**FP Relation: gamma-vinyl-GABA (D020888) - status epilepticus (D013226)**

TITLE:

Susceptibility to seizures produced by pilocarpine in rats after microinjection of isoniazid or gamma-vinyl-GABA into the substantia nigra.

ABSTRACT:

Pilocarpine, given intraperitoneally to rats, reproduces the neuropathological sequelae of temporal lobe epilepsy and provides a relevant animal model for studying mechanisms of buildup of convulsive activity and pathways operative in the generalization and propagation of seizures within the forebrain. In the present study, the effects of manipulating the activity of the gamma-aminobutyric acid (GABA)-mediated synaptic inhibition within the substantia nigra on seizures produced by pilocarpine in rats, were investigated. In animals pretreated with microinjections of isoniazid, 150 micrograms, an inhibitor of activity of the GABA-synthesizing enzyme, L-glutamic acid decarboxylase, into the substantia nigra pars reticulata (SNR), bilaterally, non-convulsant doses of pilocarpine, 100 and 200 mg/kg, resulted in severe motor limbic seizures and status epilepticus. Electroencephalographic and behavioral monitoring revealed a profound reduction of the threshold for pilocarpine-induced convulsions. Morphological analysis of frontal forebrain sections with light microscopy revealed seizure-related damage to the hippocampal formation, thalamus, amygdala, olfactory cortex, substantia nigra and neocortex, which is typically observed with pilocarpine in doses exceeding 350 mg/kg. Bilateral intrastriatal injections of isoniazid did not augment seizures produced by pilocarpine, 200 mg/kg. Application of an irreversible inhibitor of GABA transaminase, gamma-vinyl-GABA (D,L-4-amino-hex-5-enoic acid), 5 micrograms, into the SNR, bilaterally, suppressed the appearance of electrographic and behavioral seizures produced by pilocarpine, 380 mg/kg. This treatment was also sufficient to protect animals from the occurrence ofbrain damage. Microinjections of gamma-vinyl-GABA, 5 micrograms, into the dorsal striatum, bilaterally, failed to prevent the development of convulsions produced by pilocarpine, 380 mg/kg. The results demonstrate that the threshold for pilocarpine-induced seizures in rats is subjected to the regulation of the GABA-mediated synaptic inhibition within the substantia nigra.
